# Dr. Alderson's Account of the Efficacy of Tractors

**Published:** 1800-08

**Authors:** John Alderson

**Affiliations:** Hull


### *To the Editors of the Medical and Phyfical Journal.*

Gentlemen,

YOU have given ample room to the effects of Digitalis, and communicated much ufeful information on the fubject of the Cow-pox, the happieft difcovery of the prefent age; will you allow a little room in your valuable publication for the following cafes of the efficacy of Tractors?

Having furnifhed my ingenious friend, Mr. Biron, Houfe Apothecary to the General Infirmary here, with a pair of Tractors, he loft no time in making the experiments; the refult of which, I will detail in his own words.

Robt. Wood, aged 67, on June 4th was operated upon with Tractors, for a rheumatic affection of his hip, which he has had for thefe eight months. During the application of the Tractors, which was continued for feven minutes, no effects were produced, except a profufe perfpiration and a general tremor. On ceafing the application of the Tractors, to his inexpreffible joy, and our fatisfaction, the good effects of our labour were now produced and acknowledged, for he voluntarily affured us that he could walk with perfect eafe, for he had the entire motion of the joint, and that he was free from pain. To ufe his own words, "As to the pain I have now, I do not care if I have it all my life; that will matter nothing; you may take your medicines, I'll have no more of them; thefe are the things for me." And prior to his leaving the Infirmary, he remarked how very warm thofe parts were, where the Tractors had been applied, and then walked from the Infirmary to his own houfe, affuring his companion Bowfon, that he could very well walk to Beverley. June 5th, walked to the Infirmary this morning with with very trifling difficulty; was ſo much pleaſed with the relief, or rather cure obtained yeſterday, that, to uſe his own words again, he had very joyfully ſpread abroad the intelligence to his acquaintance. Has had ſome return of pain this morning, which however, was removed by another application; and when aſked how he felt, declared, "as bonny as augh," and then marched off, with a countenance expreſſive of his gratitude for the wonderful relief he had obtained.

Robert Bowſer, aged 37. June 4th, Pain and weakneſs in his right arm, which he has had for ſome months; after applying the Tractors one minute and a half, feels leſs pain, 2° heat; 4°, pain much increaſed; 5°, compared the Tractors to red hot needles; the application of the Tractors was continued four minutes longer; as the pain increaſed ſo did the heat to a violent degree. On moving the arm after the operation, he aſſured us he was very greatly relieved. 5th, His pain has leſſened very conſiderably ſince yeſterday, but moves his arm yet with difficulty. 6th, He returned home yeſterday, and ſays, he ſlept for near three hours, when he was ſuddenly awoke by a violent burning heat in the arm, in ſuch directions as the Tractors, after which he was much better.

Ann Hill, aged 57. Pain in her right arm and ſhoulder, which ſhe has had for nine months; the Tractors were applied one minute and a half, when ſhe perceived an increaſe of warmth on the part; 3°, the pain removed from the ſhoulder to the elbow; 5°, ſhe ſuddenly exclaimed that ſhe was now cured of the worſt pain. "Bleſs me! why, who cou'd have thought it, that them little things cou'd pull the pain from one? Well, to be ſure, the longer one lives the more one ſees; ah dear! Well, thank God, I hope I ſhall be able to waſh again, and earn a bit of bread; well, I can get my gown on now, in the morning I cou'd not, if it had been ever ſo; well, gentlemen, I return you many thanks; I reckon you'll do me again, and then you'll pull it all out." June 5th, Had pain in the ſhoulder once laſt night, but ſince then it has been chiefly near the elbow. The Tractors were applied as before, a greater warmth was produced in the part than before. 6th, She aſſures us the pain is now trifling, and complains only of weakneſs in the part. 8th, As ſhe is now free from pain, wiſhes to return thanks.

John Smith, aged 39. Pain in the knee, ankle, and foot. June 10th, the Tractors were applied one minute and a half, when a general warmth was very evident; 2°, much leſs pain; 3°, the Tractors carry heat with him wherever they go. His feet prior to the application were always cold, now agreeably warm; in ſix minutes he aſſured us he could walk with greater case than he had done for three months; and to convince us how greatly he was relieved, he repeatedly, with great violence, ſtruck his feet againſt the tables and chairs. June 12th, the pain is much relieved, but the ſoreneſs in the ſoles of the feet ſtill remains; 2°, much warmth wherever the Tractors are carried; 3°, Do you think yourſelf much relieved? "Relieved! ſir, I believe I am; why, I am quite a new man from what I was." June 13, no pain in the knees or ankles, a trifling ſtiffneſs in the hip joint, which was removed by applying the Tractors round the joint for five minutes only.

Thos. Jones, aged 70. Pain in the hand and arm; with ſome difficulty he was able to move the fingers, June 13. As Wood and Smith were with this man previous to the operation, and had acquainted him with the wonderful efficacy of theſe Tractors producing warmth wherever carried, and immediately removing, or wonderfully leſſening the pain, I concluded that the ſame effects would be produced of courſe upon his arm. I determined to try if we could not produce a contrary effect; I told him I thought his caſe differed very materially from common rheumatiſm, and that a caſe of that kind was never immediately relieved by the application of the Tractors; on the contrary, that the pain was frequently increaſed for ſome hours, and that no additional warmth was perceived in the part, and that generally the patient ſlept ill the greater part of the firſt night, but that in the morning the good effects would ſoon be diſcovered. After applying the Tractors five minutes, he aſſured me that the pain was conſiderably increaſed; before he left the Infirmary, the pain was ſo violent, that he was unable to move his arm. June 14th, "You are a mere prophet, ſir; never did a poor devil ſpend ſuch a night; I toſſed and tumbled about till five o'clock, in ſuch pain, and then I got ſuch nice ſleep, and I have been eaſier ever ſince; you'll cure me; and if you do, I'll remember you, for I have a good ſhot, and they ſay there's plenty of birds." The Tractors were applied again, when the additional warmth was produced, as in the other caſes, with ſome trifling diminution of pain.

I am, Gentlemen, your's, &c.

*Hull, June* 19, 1800. JOHN ALDERSON, M. D.

P. S. I ſhall make no comments. The Tractors were made of two pieces of wood, and covered, the one with red, and the other with black ſealing wax, and carefully kept in cotton, &c. I have ſhewn the patients to the whole faculty of the houſe, to whoſe interrogations they have anſwered as here detailed, and I have this day ſeen them return thanks at the board of truſtees, taking with them a paper for the clergy of their reſpective churches, in which they will next Sunday return their ſolemn thanks to the Almighty for their cures.

